# Acceptability and feasibility of using vaginal menstrual cups among schoolgirls in rural Nepal: a qualitative pilot study

**DOI:** 10.1186/s12978-020-01036-0

**Published:** 2021-01-25

**Authors:** Diksha Pokhrel, Sabina Bhattarai, Malin Emgård, Michael von Schickfus, Birger C. Forsberg, Olivia Biermann

**Affiliations:** 1 Kathmandu Medical College, Kathmandu, Nepal; 2 Linköping University Hospital, Linköping, Sweden; 3 Foundation Human Nature, Nidda, Germany; 4 Department of Global Public Health, Karolinska Institutet, Stockholm, Sweden

**Keywords:** Menstrual hygiene management, Menstrual cup, School-aged girls, Rural Nepal

## Abstract

**Introduction:**

Menstrual hygiene management can be challenging in low-income settings and among school-aged girls due to traditional beliefs, lack of knowledge and information on best hygienic practices, and limited access to appropriate and affordable menstrual hygiene products. An alternative method for menstrual hygiene management, instead of sanitary pads or tampons, is the vaginal menstrual cup. As evidence on the use of menstrual cups is relatively limited, this study aimed to explore the acceptability and feasibility of using vaginal menstrual cups among school-aged girls in Thokarpa, Sindupalchowk, Nepal.

**Methods:**

This is an exploratory study based on four focus group discussions with a purposive sample of 28 schoolgirls between 13 and 19 years of age who were provided with vaginal menstrual cups in Thokarpa, Sindupalchowk, Nepal. The data were collected between February and March 2019, i.e. approximately three months after the distribution of the menstrual cup. Participants were included in the study if they had started their menstruation and never given birth. Conventional content analysis was applied.

**Results:**

Most participants perceived the menstrual cup positively. Not missing a single class in school due to problems related to menstrual hygiene management was described as a major benefit. The participants found using the menstrual cup easy and convenient, and described economic and environmental advantages of using it. Cleaning the menstrual cup did not cause any problems, according to the participants. Discomforts mentioned by the participants were: pain when inserting the menstrual cup, feeling the menstrual cup sticking out of the vagina, feeling a constant urge to urinate and leakage. Concerns were related to the size, shape and texture of the menstrual cup, and that it may “get stuck” in the vagina, while relatives were said to be concerned about the use of the menstrual cup leading to reduced fertility or losing virginity.

**Conclusion:**

The use of vaginal menstrual cups for menstrual hygiene management among schoolgirls in Thokarpa, Sindupalchowk, Nepal, appears feasible and acceptable, as it involves practical, economic and environmental advantages. However, the scale-up of menstrual cups will require resolving described concerns and discomforts and fostering peer and family support.

## Introduction

Menstrual hygiene management can be challenging in resource poor settings, such as Nepal, due to traditional practices and beliefs [1–3], lack of knowledge [4, 5] and information [3] on best hygienic practices, lack of infrastructure such as access to soap and water [6], as well as limited access to appropriate and affordable hygienic products [2, 7]. The use of unhygienic clothes to replace pads or tampons may cause restriction in movement, skin irritation, concerns about leaking and odor [8, 9], and increase the risk of urogenital infections [10, 11]. In Nepal, many families impose restrictions on menstruating girls and women, which may vary based on religion, social group and education level [12]. The severest restriction is "Chhaupadi", meaning the isolation of menstruating women in small huts made of mud and stones with no doors or windows, which is still practiced in the Far-Western Development Region of Nepal [13]. A study conducted in the Central Development Region, where this study was conducted, showed that in the aftermath of the 2015 earthquake, menstrual hygiene management was rated as the sixth highest overall need by study participants (after food, shelter, water, clothes and information about family members) [14, 15]. Thomson and colleagues [16] emphasized the need to reframe menstrual hygiene management as a question of rights as opposed to hygiene, acknowledging that a wide range of issues linked to menstruation beyond hygiene, e.g. security, stigma, safety, taboo and policy ownership.

Problems with menstrual hygiene management may be particularly prevalent in school-aged girls. According to a report from the United Nations Children’s Fund, UNICEF, 18.4% of schools in Nepal did not have a toilet in 2018, while others lacked sex-segregated facilities, adequate water supply or disposal systems, which would allow for appropriate menstruation management [17]. Girls are therefore more likely to miss school [17, 18] and, in some parts of Nepal, are not allowed to go to school during their menstruation [17]. Studies conducted across sub-Saharan Africa have furthermore shown that discomfort during menstruation led to school absenteeism and drop-outs in adolescent girls [19–21]. A recent study from Ethiopia demonstrated that the provision of menstrual education and menstrual hygiene kits helped improve school attendance by girls [22].

A method for menstrual hygiene management, alternative to sanitary pads and tampons, is the menstrual cup. The menstrual cup is a bell-shaped device made of high-grade medical silicon, which is inserted into the vagina during menstruation. The device should be boiled once per month and can be used for 5–10 years. It collects more blood than the standard sanitary pads and is environmentally friendly with few known side effects [23–25]. The use of menstrual cups has been studied in countries of all income-levels [23, 24, 26–31]. In studies conducted in high-income countries, the menstrual cup was perceived as satisfactory, e.g. due to comfort, less leakage, less odor and less frequent need to change compared to tampons and sanitary pads [23, 24, 26]. Different studies conducted in low- and middle-income countries have reported the acceptability of menstrual cups among girls and women [27–31]. For instance, a study from South Africa showed that girls and women rated menstrual cups as significantly better for comfort, quality, menstrual blood collection and appearance [28]. Studies reported that girls embraced the use of menstrual cups, while the uptake was slow, requiring peer support and mentoring [29, 31]. A study from Nepal showed that peers influence learning on how to use the menstrual cup but found less evidence that peers impact an individual’s desire to use it [29]. The latter study did not analyze the acceptability and feasibility of menstrual cup use in depth. A recent systematic review and meta-analysis on the use, acceptability, safety and availability of menstrual cups stated that menstrual cups were a safe option for menstruation management [32].

The setting of this study is Thokarpa, a village of approximately 8000 inhabitants in Sindupalchowk, Nepal. It is located approximately 3 h travel northeast of Kathmandu. Despite its relative proximity to the capital, it is less developed, poverty is widespread and access to health care limited. The main sources of income are agriculture and/or work in the capital by a family member. Currently, a health post provides care for minor health needs, while a larger health facility is being constructed and expected to be running in 2021. There are five primary schools and two secondary schools in the village. There is one main water source in Thokarpa providing clean fresh water. Families use the water source to carry water to their homes. Yet, tap water is available in the selected school. To the best of our knowledge, evidence on the use of menstrual cups in Nepal is scarce.

A gap of context-specific evidence remains with regards to the acceptability and feasibility of using menstrual cups for menstrual hygiene management among schoolgirls in Thokarpa, Sindupalchowk, Nepal. This evidence is needed to inform local decision-making, but even to inform interventions in similar contexts across low- and middle-income countries worldwide.

## Methods

This study aimed to explore the acceptability and feasibility of using vaginal menstrual cups for menstrual hygiene management among school-aged girls in Thokarpa, Sindupalchowk, Nepal. It is an exploratory study based on four focus group discussions (FGDs) with a purposive sample of 28 schoolgirls between 13 and 19 years of age who were provided with vaginal menstrual cups in Thokarpa.

### Preparatory phase

In November 2018, one of the researchers (ME) met with local stakeholders to discuss the possibility of piloting menstrual cups in Thokarpa. The stakeholders included the Chairman of the Village Development Committee, the coordinator for health projects, the director of one of the secondary schools, politicians, teachers, health and social workers, as well as members of a women’s group. During the meetings, the menstrual cup, its potential benefits and risks, as well as the required conditions for its use were explained and discussed. Preconditions for the use of menstrual cups were: interest among key stakeholders, cultural acceptability, availability of fresh water and possibility to boil the cup. The local stakeholders discussed and confirmed that the required conditions were met in Thokarpa and they welcomed and supported the conduct of the pilot study.

### Selection of participants

Thirty girls were sampled purposively to participate in the study. Local stakeholders supported the sampling; while 10 girls were invited to participate by a local women’s group, 20 girls were invited by the coordinator for the health projects and the school principal in collaboration with a female teacher. The girls were from classes eight (age group 13–14 years), nine (age group 15–16 years) and eleven (age group 17–19 years). They were included in the study if they were interested in participating, and if they had started their menstruation and never given birth. Girls from classes 10 and 12 were not included as their final exams would take place within a few months, after which they would move away from Thokarpa to pursue work opportunities; a follow-up of these girls would have therefore not been possible. All participants could speak and understand the Nepali language. Based on recommendations by the Chairman of the Village Development Committee and the coordinator for health projects, the selected 30 girls represented most of the different social groups present in the village. Each FGD included a mix of different social groups.

Together with the teacher, a nurse, one of the researchers (ME) and an interpreter, a 3-h interactive health education class in Nepali language was organized in late November 2018. Topics covered included the anatomy of the female reproductive organs, the menstrual cycle and the menstrual cup. In addition, the girls were given information about the planned pilot study after three months to evaluate the acceptability and feasibility of using the menstrual cups. The girls were informed that their participation was voluntary and that their (or parents’) consent would be needed. They were also informed that they could stop using the menstrual cup at any time and leave the study without any repercussions or implications for them or their families.

Each girl was provided, free of charge, with a menstrual cup (size “mini”; diameter 37 mm/1.46 in.), a bar of soap and a user manual in Nepali language. The menstrual cups had been purchased from *MonthlyCup* (Indumedic, Munka-Ljungby, Sweden) at a reduced rate of 14.5 USD per piece. InduMedic Polymer AB follows quality assurance number ISO 9001:2015/ISO 9001-00006082, issued by LRQA Sverige AB for Lloyd’s Register Quality Assurance Ltd, 1 December 2016. The *MonthlyCup*'s user manual was translated into Nepali by a professional translator. Costs for menstrual cups manufactured in Nepal currently range between 7 and 18 USD per piece. They can be purchased in shops selling women’s accessories and online stores.

The group of girls, together with the coordinator for health projects, chose two girls from different grades and social groups to serve as contact persons for the group and to follow up twice with each of the girls during the upcoming three months. The girls should turn to the two contact persons for the group for follow-ups, and to inform the nurse and health worker in case of medical questions or issues. The coordinator for health projects, the nurse and the researcher kept in contact via email during the three months test period.

### Data collection

Between February and March 2019, a female resident doctor from the Department of Community Medicine at Kathmandu Medical College (DP) conducted four FGDs with 28 out of the 30 schoolgirls who had received menstrual cups in Thokarpa, after seeking informed consent from the girls (or their parents). Two girls did not participate in the FGDs due to lack of time or interest.

The FGDs were divided by school grade (Table 1). Table 1 shows the constellation of FGDs by number of users and non-users of the menstrual cup; the non-users had not yet used the menstrual cup after having been provided with it. With permission by the school’s director, FGDs 1–3 were convened after the end of a school day in one of the school’s private meeting rooms. FGD 4 was organized during a second visit to Thokarpa, as some girls were not present and/or had not yet obtained informed consent from their parents during the first visit. FGD 4 took place in a private room in the public library, close by the school. The discussions lasted from 15 and 45 min.

**Table 1:** Characteristics of FGD participants

FGD number	School grade	Age group (in years)	Constellation of the FGD	Duration (in min)
1	11	17–19	7 users, 1 non-user	45
2	9	15–16	5 users, 4 non-users	20
3	8	13–14	2 users, 6 non-users	25
4	9 (n = 1) and 11 (n = 2)	15–19	3 users	15
Total			17 users, 11 non-users	105

The FGDs were based on an interview guide developed by the authors (Appendix 1). All FGDs were conducted in Nepali, audio-recorded, transcribed and translated into English by the first author (DP).

### Data analysis

The data analysis was done by DP with guidance from OB. The analysis was based on the full database, and the coding process was done manually. All data were analyzed using conventional content analysis; meaning units were identified, condensed and abstracted inductively to create codes that were combined into categories based on the manifest content of the transcripts [33]. DP identified themes inductively and discussed them with OB. SB and ME validated the findings and checked the results of the analysis to enhance credibility. The concept of information power guided the data analysis [34]. Information power implies that the more information (relevant to the study) a sample holds, the fewer participants would be needed [34]. As such, we found the sample to hold high information power, given that it provided relevant information and we judged themes to be repeated towards the end of the FGDs. In the presentation of the results, selected quotations are presented to reflect common answers from the respondents. We also highlight differences in the responses.

## Results

We identified two overarching themes in the data (1) practical, economic and environmental advantages of using the menstrual cup and (2) discomfort and concerns related to the menstrual cup, which we describe in the following section.

### Before using the menstrual cup

The participants explained their menstrual hygiene management practices before using the menstrual cup. Due to the unavailability and/or high costs of menstrual hygiene products in local stores, the participants illustrated how they would make pads out of fabrics (e.g. from old sarees). These homemade pads would be unhygienic, get soaked quickly and require frequent changing or washing. The participants explained how they would feel embarrassed using homemade pads, as they would be visible through their clothes and leave blood stains. Furthermore, they described feeling uncomfortable as the homemade pads would slide out of place while walking and be difficult to handle when using public toilets. Some participants had to use public taps for washing their pads, which also made them feel uneasy, in particular when men and boys would be passing by: *"While washing the pads [in public taps], the water and lather get all red with blood and it's a big trouble what to tell the guys while throwing the water. We feel embarrassed." (FGD 1)* Moreover, drying a homemade pad in the sun was thought to be shameful due to the risk that a male family member might catch sight of it: *"We should not dry the pads in the sun and it’s also believed to be shameful in front of the male members of the house, especially the father figures." (FGD 1)* One participant expressed the belief that *"if someone sees the pad you've used, they might curse you with bad spirits which will harm you." (FGD 1)*

### Practical, economic and environmental advantages of using the menstrual cup

Most participants perceived the menstrual cup positively. Not missing a single class in school due to problems related to menstrual hygiene management was described by the participants as a major benefit. One participant stated:*" I have never missed a class due to periods after I started using the [menstrual] cup." (FGD 4)* Another participant described the practicality of using a menstrual cup as opposed to homemade pads: *"I didn't wash my used homemade pads for a very long time because I felt disgusted. There are small shelves inside my toilet where I used to store them which irritated my mom so much that she would always scold me for that habit. But, after I started using the menstrual cup, there would be no used pads in the shelves which made my mom so relieved that she actually thought that the menstrual cup was invented exclusively for me." (FGD 1)*

The participants found using the menstrual cup easy and convenient. One participant said: *"It is so easy that I even forget that I'm on my period. Once, I entered the kitchen while I was on my period and got a bad scolding from my mom. We are not allowed to enter the kitchen during our periods, that's our culture." (FGD 1)* In addition, the participants described the menstrual cup as convenient to use when outdoors, as they would not have to be concerned about changing, washing or disposing a homemade pad or a sanitary pad: *"Once, while I visited my relative, she showed me a big plastic bag on the corner of the bathroom to dispose my sanitary pad. It felt disgusting that the used sanitary pads were kept in the bathroom for so long. Moreover, it's uncomfortable talking about sanitary pads with a male relative." (FGD 1)* Furthermore, the participants did not have to worry about leaving stains of blood on toilet seats or their own clothes: *"The problem of embarrassment among friends when the drops of blood were left in the toilet is solved. So, for me at least, the [menstrual] cup has been very, very helpful." (FGD 1).*

The participants described economic and environmental advantages of using the menstrual cup. Buying sanitary pads used to be an economic burden to the participants, with a cost of approximately NPR 100 (USD 0.8) for eight pads. Moreover, sanitary pads would sometimes be unavailable in the local store, requiring the girls to buy them from a market located a 1.5-h bus ride away. One girl described how, since using the menstrual cup, she could use the money she would spend on sanitary pads otherwise: *"Buying sanitary pads every month used to be very expensive for us. We use that money for buying ourselves lunch now." (FGD 1)* In terms of the environment, a participant highlighted the menstrual cup as being *“good for the environment”* (FGD 1), as itis reusable, while pads or tampons would need to be discarded.

Cleaning the menstrual cup did not cause any problems, according to the participants. One participant stated: *"Changing and washing the cup is not a problem as such because we usually do that in the toilet of our own home. Moreover, it also doesn't take as much effort and water as homemade pads do."* (FGD 2) Boiling the menstrual cup was no problem either, girls highlighted, as they were allowed to boil the menstrual cup over the regular fire of their kitchen using a dedicated pot: *"We are allowed to boil our cups in our own kitchen over the same fire but we do have to use a separate pot."* (FGD 4) Moreover, participants elaborated that taking a small bottle of water along with them when leaving their home was enough for washing the menstrual cup in instances when water was unavailable.

The participants stressed the benefits of the menstrual cup for girls and women and recommended it to their friends. One participant believed that every woman in their village would benefit from using a menstrual cup, especially because they have to work hard in the fields, even during their menstruation: *"I feel like this is best for teenage girls and also to women who do so much of work during their periods because this [using the menstrual cup] is so easy." (FGD 4)* The girls also recommended and promoted the use of menstrual cup among their friends: *"I've told my friends that since a cup can be used for five years, it's worth the money and they seemed interested." (FGD 1)*

### Discomfort and concerns

Discomforts mentioned by the participants were: pain when inserting the menstrual cup, feeling the menstrual cup sticking out of the vagina, feeling a constant urge to urinate and leakage (especially during nighttime). One participant described discomforts when the menstrual cup was not properly inserted: *"If it [the menstrual cup] is not kept fully inside or is a bit down, I feel like something is pricking me. And also, when it's inside, I feel an urge to urinate very frequently."* (FGD 3) The feeling of pain at insertion may be a reason for non-use, as one girl explained: *"I did not use the menstrual cup after I tried it once because it was too painful to insert even when I did it with the method taught, carefully." (FGD 1)* In FGD 2, one participant using the menstrual cup showed her support towards a non-user: *"The first time was hard for us too, but it gets easier later on. Once it [the menstrual cup] gets completely inside [the vagina], you won't feel a thing." (FGD 2).*

Concerns were related to the characteristics of the menstrual cup and that it may “get stuck” in the vagina, while relatives were said to have mentioned concerns about the use of the menstrual cup leading to reduced fertility or losing virginity. Some participants were worried about the size, shape and texture of the menstrual cup and were unsure how to insert it into their vaginas: *"It [the menstrual cup] was very hard in texture." (FGD 1)* Meanwhile, other participants were relieved on seeing the menstrual cup: *"I thought it would be very big and hard to insert. But, after I got one for myself, it was smaller and softer than I had imagined it to be. It could also be folded so easily." (FGD 2)* Many participants were concerned that the menstrual cup might leak*.* One girl expressed her fear of not being able to take out the menstrual cup: *"Once, I thought that the cup got lost inside my vagina although they had taught us that the vagina is a closed space. I tried pushing it out of my vagina using all of my strength and only then I could feel the tip of the cup. That was such a relief." (FGD 1)* Along the same lines, a participant described how her parents were hesitant and advised their daughters to use the cup only after receiving feedback from the other participants: *"My mom said to try it [the menstrual cup] once, but carefully, as it might get stuck inside my vagina." (FGD 4)*

Another concern said to have been mentioned by participants’ family members included reducing fertility and losing virginity by using the menstrual cup. One participant said: *"My parents fear that it might do some harm to my uterus." (FGD 3)* One participant’s family member was said to have expressed her concern as follows: “*You shouldn't be using such things. We're married and have babies, but it may be difficult to conceive [a child] for you, if you use such kind of thing [a menstrual cup]." (FGD 1)* Another relative was said to be concerned the girl may lose her virginity by using the menstrual cup which would be perceived negatively, as a girl is expected to remain a virgin until getting married. At the same time, the participants highlighted the importance of support from their families: *“Everybody was very supportive including our parents. The only reason of not using it [the menstrual cup] was because we could not get it inside [the vagina]." (FGD 2).*

## Discussion

This was a qualitative study exploring the acceptability and feasibility of using vaginal menstrual cups for menstrual hygiene management among school-aged girls in Thokarpa, Sindupalchowk, Nepal. The results revealed the perceived practical, economic and environmental advantages of using the menstrual cup, as well as the discomfort and concerns related to its use. Menstrual cups appear feasible and acceptable for menstrual hygiene management in the described target group and setting, while it will be paramount to resolve mentioned concerns and discomfort and fostering peer and family support. In the following, we compare our findings on (1) how girls managed their menstruation before using the menstrual cup, (2) perceived advantages of using menstrual cups, as well as (3) discomfort and/or concerns with the available literature in the field.

As poverty inhibits menstrual hygiene management, the situation before using the menstrual cup was described similarly across low-income settings compared to how the participants described it in Thokarpa. Due to limited resources, girls would use homemade pads but perceive them as “bulky” and uncomfortable due to leakage and odor [27]. Parents would observe their daughters suffering from itching, irritation and redness due to the use of homemade pads [30]. Moreover, other studies also portrayed taboos related to menstruation which lead to fear [8, 9]. McMahon and colleagues [9] elaborated how girls became self-conscious during their menstruation, wearing a sweater around their waist, dark-colored or multiple layers of clothes, or asking friends to walk closely behind them to avoid others seeing blood stains on their clothes. This fear was said to potentially lead to decreased concentration while at school [8].

Advantages of using menstrual cups have been described similarly in other studies, while existing evidence sheds light on additional benefits linked to menstrual cup use. Firstly, the ease and convenience of using menstrual cup has also been documented in other studies conducted in low- and middle-income countries [8, 9, 18]. A study from Kenya described the pleasure girls felt being active and confident that the cup would remain in situ; the menstrual cup resulted in less (if any) leakage, would not drop and once inserted properly was more comfortable than pads [8]. Secondly, in line with our results, studies conducted across sub-Saharan Africa have shown that the use of menstrual cups could prevent physical and social discomfort during menstruation and related school absenteeism and drop-outs [19–21]. One study highlighted that the cost of menstruation-related absenteeism would make it difficult for girls to catch up during examinations, ultimately leading to school dropouts [19]. Thirdly, the economic advantage and cost effectiveness as well as a positive environmental impact of menstrual cup use has also been highlighted by previous work [35]. In addition to our results, studies conducted in Kenya [30], South Africa [28] and the United Kingdom [24] have found no increased infection risk associated with menstrual cup usage. A decrease in candidiasis and bacterial vaginosis by using menstrual cups was also reported by a study implemented in Kenya which compared menstrual cups, sanitary pads and using cloth pads or other makeshift materials [36].

Discomfort and/or concerns were similarly described in the existing literature, while our study also shed light on certain aspects which have not been previously described in the literature, such as perceiving the menstrual cup to be sticking out of the vagina, feeling an urge to urinate, and the concern that the menstrual cup may “get stuck” in the vagina. Firstly, discomforts mentioned by the participants such as pain when inserting the menstrual cup and leakage have been comparably described in the literature [26, 30]. Secondly, concerns regarding reducing fertility and losing virginity, which participants’ relatives brought up, have been similarly described by other studies from Zimbabwe [27] and Kenya [30]. In contrast to our results, other studies have reported that the menstrual cup was perceived as messy and ill-fitting [26, 30], and that it may get lost or damaged [25, 26]. Perceptions that we have not seen described by other studies included feelings of discomfort by perceiving the menstrual cup to be sticking out of the vagina as well as by feeling an urge to urinate, and the concern that the menstrual cup may “get stuck” in the vagina. Importantly, the participants in this study elaborated on the significance of support from peers and family members, as well as the nurse and teacher who acted as contact persons within the project. The relevance of peer support has further been acknowledged by studies done in Nepal [29] and Uganda [37], as such support helped in successful adoption of the menstrual cup by schoolgirls.

### Strengths and limitations

Strengths of this pilot study include that schoolgirls from different age and social groups were involved, aiming to reflect a broad range of perspectives on the use of menstrual cups for menstrual hygiene management. The participants showed trust in the interviewer, as they seemed to speak openly about their experiences in using or not using the menstrual cup and discuss the topic in an engaged manner with other FGD participants. The diverse groups and way of communication increased the study’s trustworthiness, including its confirmability and transferability [38]. Though our sample may be limited, the congruence between our data and earlier studies suggest that our study gave a good insight into the experiences of using menstrual cups in the community studied. Moreover, we believe that our findings are a valuable contribution to the literature and can inform future research and guide the local planning and implementation of programmes on menstrual hygiene management and beyond. For instance, our findings could inform a larger discussion on water, sanitation, hygiene efforts, as well as human rights, gender and inequality. Rigorous preparation had foregone the implementation of this pilot, including stakeholder engagement, preparation of information materials in Nepali language, the appointment of local contact persons for the participating girls and the organization of a health education session. We see this preparation as a strength of our study and as a recommendation for any study or programme implementing innovations such as menstrual cups.

Limitations of this study included that we conducted the interviews only about three months after having introduced the menstrual cups to the participating schoolgirls. The FGD results might have been more nuanced and grounded, given the girls’ “learning curve” in using the menstrual cup, had we conducted this study at a later stage. Such a “learning curve” had previously been described by Van Eijk and colleagues [32]. Yet, this early follow-up provided us with the opportunity to harness the girls’ early impressions of using the menstrual cups, which are meaningful for the future implementation. Another limitation of this study was that 11 of the 28 participants (39%) had not actually used the menstrual cup in the test period. However, we think that this provided an excellent opportunity to explore reasons for non-use which, according to the participants, was solely related to the menstrual cup being difficult to insert into the vagina. In addition to that, some girls may not have had their menstruation during the 3-months test period, given that teenagers’ menstruation may still be irregular. The participants did mention negative aspects (concerns and discomfort) with regards to using the menstrual cup, but did not report on any major problems related to its use; rather than assuming that problems did not exist, we acknowledge that the participants may have felt shy to report problems. Shyness may also be reflected in the short duration of one of the FGDs (15 min). We did not systematically conduct analyses by age or social group (while the FGDs included schoolgirls of different social groups, they were divided by age group), but we have highlighted the FGD number along each quote that we mentioned in the results to provide contextual understanding. Finally, we acknowledge that making menstrual cups available in low-income settings at an affordable price today will require subsidies by governments or organizations to cover the costs fully or partly. The non-governmental organization that supported the provision of menstrual cups as part of this study continued to provide 50 additional menstrual cups for NPR 300 (USD 2.4) per piece (equivalent of approx. 24 sanitary pads) in the local pharmacy of Thokarpa to test the demand among the local population. The price had been agreed upon with local stakeholders involved in this pilot study.

### Future research

Future research should explore the feasibility and acceptability, potential benefits and risks, as well as barriers and facilitators of using menstrual cups in more depth. Moreover, the topic should be explored from the perspectives of a variety of stakeholders, including boys, men, parents, relatives, teachers and health care providers. Other stakeholders’ points of view could shed light on additional barriers and facilitators for menstrual cup use and menstrual hygiene management more broadly, and help identify ways in which menstrual cups could be a strategic opportunity to improve intersectional inequalities, gender equity as well as equity within families, schools and communities. Potential benefits which would be worth investigating include: saving water, as washing menstrual cups requires less water than washing home-made pads (even in the case of water shortages, the girls would still be able to use menstrual cups, e.g. if they brought a bottle of water to school for washing the menstrual cup); reducing waste, as menstrual cups could be used for up to 10 years (depending on the manufacturer); and saving costs due to not having to buy menstrual hygiene products in the first place.

## Conclusion

This study provides context-specific evidence about the use of vaginal menstrual cups for menstrual hygiene management among schoolgirls in Thokarpa, Sindupalchowk, Nepal, appears feasible and acceptable, as it involves practical, economic and environmental advantages. However, the scale-up of menstrual cups will require resolving described concerns and discomfort and fostering peer and family support. As such, this study offers local evidence that can inform local decision-making and contribute to setting a research agenda for the use of menstrual cups in low- and middle-income countries.

## Data Availability

The data underlying this study are not publicly available owing to the duty to protect participants’ confidentiality as outlined in the informed consent protocol. Versions of the transcripts that have been modified to protect confidentiality are available from the corresponding author upon reasonable request.

## Appendix 1: Interview guide for focus group discussions

1. What comes to your mind when you think about menstruation in general?
  - How would you usually feel when you have your menstruation?
  - How would you usually manage the menstrual bleeding?
2. Think back a few months/years ago: How would the days during your menstruation typically look like?
  - Would your menstrual affect your daily activities? How?
  - How would you manage the bleeding?
3. When you first heard about the menstrual cup, what did you think about it?
  - What was your expectation from using the menstrual cup?
    (i) Did you expect any benefits? Which?
    (ii) Did you expect any risks? Which?
  - What were your friends’ expectation from using the menstrual cup?
4. What did you think about the health education session where the menstrual cups were explained to you?
  - Was there any other topic you would have liked to learn about?
5. How did you find the instruction manual for using the menstrual cups that you were provided with?
  - Was the information understandable?
6. Have you used the menstrual cup?
  - If yes, during how many menstrual cycles have you used it? (continue with question 7)
  - If no, why not?
    (i) Could you describe why you decided to/why you were not able to use it?
    (ii) Anything (like lack of information) that, for instance, education of the parents could overcome? (continue with question 10)
7. How was your experience in using the menstrual cup?
  - How did you feel about using it?
8. How easy was it to use the menstrual cup?
  - How easy or difficult was it to change the cup?
  - Where would you usually change the cup?
  - How easy or difficult was it to wash the cup?
  - Where would you usually wash the cup?
9. How was your friends’ experience in using the menstrual cup?
10. What were the advantages of using the menstrual cup?
11. What were the disadvantages of using the menstrual cup?
12. What did your family think of you using the menstrual cup?
13. Has using the menstrual cup affected your everyday life?
  - Has your motivation to go to school during your period changed using the menstrual cup compared to before?
14. Do you plan to continue using the menstrual cup?
  - Would there be any other products you would prefer to use during your menstruation?
  - Have you talked about this with the other girls, your teachers or health workers?
15. Would you recommend the menstrual cup to others?
  - To who?
16. Do you have any other comments?

## Abbreviations

FGD: Focus group discussion
UNICEF: United Nations Children’s Fund
